# Retraction: Salvage Liver Transplantation for Patients with Recurrent Hepatocellular Carcinoma after Curative Resection

**DOI:** 10.1371/journal.pone.0220394

**Published:** 2019-07-23

**Authors:** The PLOS ONE Editors

Concerns have been raised that the transplants performed in the local context at the time of procedures reported in this article [1] may have involved organs/tissues procured from prisoners [2].

This article [1] reports a study that analyzed data for patients who received liver transplants from deceased donors between 2004–2008, but details as to the donor sources and methods of obtaining donors’ informed consent were not reported. In follow-up discussions about transplant donor sources and consent, the authors clarified that most organs for transplant procedures performed at the Organ Transplantation Center, First Affiliated Hospital of Sun Yat-sen University from 2004–2008 were procured from executed prisoners. The authors did not provide documentation when requested by the journal to confirm that the study had institutional ethics approval. In addition, the authors did not respond to inquiries about the availability of underlying data supporting this study.

International ethics standards call for transparency in organ donor and transplantation programs and clear informed consent procedures including considerations to ensure that donors are not subject to coercion. Owing to the lack of documentation to demonstrate this study had prospective ethical approval, insufficient reporting, concerns about the use of prisoners as transplant donors, and unresolved concerns around the informed consent procedure used for organ donors, and in compliance with international ethical standards for organ/tissue donation and transplantation, the *PLOS ONE* Editors retract this article.

The first author notified the journal that all authors agree with the retraction. The other authors either could not be reached or did not respond directly.
